# Transcriptional profiles in *Strongyloides stercoralis* males reveal deviations from the *Caenorhabditis* sex determination model

**DOI:** 10.1038/s41598-021-87478-3

**Published:** 2021-04-15

**Authors:** Damia Gonzalez Akimori, Emily J. Dalessandro, Thomas J. Nolan, Christopher R. Stieha, James B. Lok, Jonathan D. C. Stoltzfus

**Affiliations:** 1 Department of Biology, Millersville University of Pennsylvania, Millersville, PA 17551 USA; 2 Department of Pathobiology, School of Veterinary Medicine, University of Pennsylvania, Philadelphia, PA 19104 USA

**Keywords:** Parasitology, Parasite biology, Parasite development, Parasite evolution, Parasite genetics, Parasitic infection

## Abstract

The human and canine parasitic nematode *Strongyloides stercoralis* utilizes an XX/XO sex determination system, with parasitic females reproducing by mitotic parthenogenesis and free-living males and females reproducing sexually. However, the genes controlling *S. stercoralis* sex determination and male development are unknown. We observed precocious development of rhabditiform males in permissive hosts treated with corticosteroids, suggesting that steroid hormones can regulate male development. To examine differences in transcript abundance between free-living adult males and other developmental stages, we utilized RNA-Seq. We found two clusters of *S. stercoralis*-specific genes encoding predicted transmembrane proteins that are only expressed in free-living males. We additionally identified homologs of several genes important for sex determination in *Caenorhabditis* species, including *mab-3*, *tra-1*, *fem-2*, and *sex-1*, which may have similar functions. However, we identified three paralogs of *gld-1*; *Ss-qki-1* transcripts were highly abundant in adult males, while *Ss-qki-2* and *Ss-qki-3* transcripts were highly abundant in adult females. We also identified paralogs of pumilio domain-containing proteins with sex-specific transcripts. Intriguingly, *her-1* appears to have been lost in several parasite lineages, and we were unable to identify homologs of *tra-2* outside of *Caenorhabditis* species. Together, our data suggest that different mechanisms control male development in *S. stercoralis* and *Caenorhabditis* species.

## Introduction

*Strongyloides stercoralis* is a skin-penetrating parasitic nematode that infects approximately 614 million people globally and causes the disease strongyloidiasis, which can persist for decades and is often asymptomatic^1^. However, *S. stercoralis* infection can result in chronic intestinal and/or respiratory issues, as well as death in cases of hyperinfection and dissemination, which can be triggered by either corticosteroid treatment or human T-lymphotropic virus type 1 (HTLV-1) co-infection^2^. *S. stercoralis* also infects dogs and non-human primates^3^, and the presence of genetically similar strains in dogs and humans indicate the potential for zoonotic transmission^4^. *S. stercoralis* is used as a model to study nematode parasitism, since it is one of the more tractable species for genetic manipulation due to the ability to insert transgenes in the free-living generation^5^.

The *S. stercoralis* life cycle is more complex than other obligate parasitic nematodes and can alternate between a parasitic and a single gonochoristic (with distinct male and female individuals) free-living generation^3^. The parasitic female, which lives in the crypts of the small intestine^3^, reproduces by mitotic parthenogenesis^6^. The post-parasitic generation, which can include both males and females^7,8^, develops either homogonically to infectious larvae or heterogonically to free-living adults^3^. Post-parasitic males invariably develop into free-living rhabditiform (short pharynx with two bulbs) larvae. By contrast, post-parasitic females can either develop into free-living rhabditiform larvae or dauer-like filariform (elongated and radially constricted pharynx) infectious third-stage larvae outside of the host, termed iL3, or inside the host as autoinfective larvae, termed aL3^3^. Free-living adult males and females reproduce sexually^9^, producing post-free-living larvae that are all female and invariably develop into iL3^10^. For iL3 to continue development, they must infect a permissive host—where they eventually mature into parasitic females^3^.

The switch controlling homogonic versus heterogonic development for *S. stercoralis* post-parasitic female larvae is triggered early in the first larval stage (L1)^11^. Factors controlling this switch include temperature^12^ and strain genetics^13^, and these signals are mediated, in part, by dafachronic acids^14^. By contrast, post-parasitic male development appears to be determined by the allocation of a single X chromosome to the egg^12^. Greater than 95% of post-parasitic larvae in the *S. stercoralis* strain used in this study develop via the heterogonic route when cultured at 22 °C.

Although most nematodes, including the model organism *Caenorhabditis elegans*, utilize an XX/XO mechanism for sex determination, other mechanisms have evolved, including Y chromosomes in some filarial species^15^ and chromosomal diminution in some *Strongyloides* species^16,17^. Furthermore, significant plasticity has been observed in nematode sex chromosomes, as new sex chromosomes have evolved in several lineages^15^. Sex determination in *S. stercoralis* is likely an XX/XO mechanism, similar to the closely-related *S. ratti*^18^. Both *S. stercoralis* and *S. ratti* have two pairs of autosomes, with free-living and parasitic females additionally possessing two X chromosomes (2n = 6) and males additionally possessing one X chromosome (2n = 5)^6,18^. However, in *S. papillosus* and *S. vituli*, chromosome I and X have fused, and males undergo a sex-specific diminution of the X-containing portion for just one of these fused chromosomes^16,17^. Since the more distantly related facultative parasite *Parastrongyloides trichosuri* has three pairs of chromosomes and also utilizes an XX/XO sex determination system, chromatin diminution in *S. papillosus* and *S. vituli* is likely a derived trait^17^. In contrast to *C. elegans*, where the X chromosome to autosome ratio is the initiating sex determination signal, environmental signals are sensed by the parasitic female and control the elimination of an X chromosome (in the case of *S. stercoralis* and *S. ratti*) or the portion of a chromosome (in the case of *S. papillosus* and *S. vituli*) and thus male development in *Strongyloides* species^19^.

The proportion of males and females in the post-parasitic generation is unequal in *Strongyloides* species, and the proportion of free-living males increases over the duration of an infection for both *S. stercoralis* and *S. ratti*^12,20^. Furthermore, immunosuppressing the host can decrease the proportion of larvae developing into free-living males in *S. ratti*^20^. The molecular mechanism behind this environmentally-influenced sex determination system is unknown. However, previous studies have hypothesized that *S. ratti* may use a modified form of mitotic parthenogenesis where only X chromosomes undergo a “mini meiosis,” resulting in XO oocytes and XXX polar bodies^18^. Chromosomal non-disjunction is also a possibility; however, this mechanism results in both XO male and XXX progeny in *C. elegans*^21^, and XXX progeny have not been observed in *Strongyloides* species (although they could be non-viable).

The mechanism by which post-free-living larvae invariably inherit the paternal X chromosome, in addition to a maternal X chromosome, is also unclear. One possibility is that spermatids receiving an X chromosome attract the bulk of the cytoplasm and organelles required for sperm function, similar to that observed in the trioecious nematode *Auanema rhodensis*^22^. In *S. papillosus*, mature sperm with a diminished X region are not formed^16^; however, nullo-X sperm have been observed in *S. ratti*^23,24^, and meiotic cells with either two or three chromosomes have been observed in the male testis of *S. stercoralis*^6^. In both *S. ratti* and *S. stercoralis*, free-living males contribute genetic material to the offspring, ruling out pseudogamy^9,25^.

**Figure 1:**
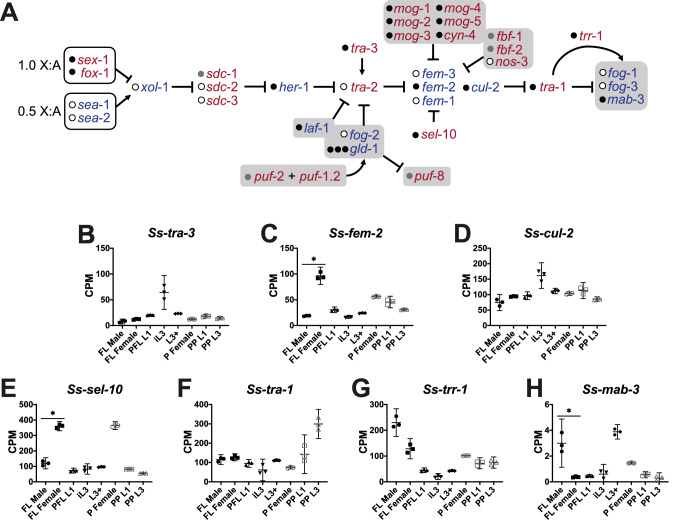
Components of the *Caenorhabditis* sex determination pathway are present in *S. stercoralis*, and key transcription factors likely have conserved function. (**A**) Genes that compose the “core” sex determination pathway in *Caenorhabditis* species, along with accessory genes that play a role in somatic (white boxes) and germline (gray boxes) sex determination, are diagrammed, with feminizing factors in red and masculinizing factors in blue. Homologs in *S. stercoralis* (black circle), homologs encoding a similar type of protein (gray circle), and genes for which no homolog was identified in the *S. stercoralis* genome (white circle), are indicated. Adapted from^26^. (**B**–**H**) Transcript abundances of the *S. stercoralis* homologs are represented using TMM-normalized counts per million (CPM) for the following developmental stages: free-living adult males (FL Male), free-living gravid adult females (FL Female), post-free-living first-stage larvae (PFL L1), developmentally arrested infectious third-stage larvae (iL3), L3 activated inside a permissive host (L3 +), parasitic gravid adult females (P Female), heterogonically-developing post-parasitic L1 (PP L1), and heterogonically-developing post-parasitic L3 enriched for females (PP L3). Graphs were constructed using GraphPad Prism v.9.0.0; bars indicate means (horizontal) and 95% confidence intervals (vertical) for each of the three biological replicates represented as individual data points. Asterisks (*) indicate a significant (fold change > 2.0; FDR < 0.05) difference between FL Male and FL Female.

*Caenorhabditis* species have several genes that detect the ratio of autosomes to X chromosomes, genes that propagate this signal, and genes involved in tissue-specific responses for both somatic and germline sex determination (Fig. 1A). A few of these genes, including the transcription factors encoded by *mab-3* and *tra-1*, appear to regulate sex determination in a broad range of species; however, there is some divergence in genes controlling sex determination at the periphery of the “core” pathway^26^ (Fig. 1A). As proposed by Wilkins (1995), the “bottom up” hypothesis posits that the most downstream genes in a sex determination pathway evolved before upstream elements and, therefore, should be more conserved between nematode species^27^.

The genes important for regulating the development of *Strongyloides* (clade IV^28^) free-living males and females, or their gametes, are not well-understood. Additionally, whether homologs of genes important for sex determination in *Caenorhabditis* species (clade V) have been conserved and have similar functions in *Strongyloides* species is also unknown. We therefore sought to identify *S. stercoralis* homologs of genes involved in sex determination and characterize the differences in gene expression in free-living adult males and females.

## Materials and methods

### *S. stercoralis* maintenance and experimental infections

We maintained *S. stercoralis* strain PV001^29^ in prednisolone-treated beagles, as previously described^30^, in accordance with protocols 702342, 801905, and 802593, approved by the University of Pennsylvania Institutional Animal Care and Use Committee (IACUC). We conducted experimental infections of *Meriones unguiculatus* (Mongolian gerbils) strain Crl:MON(Tum) (Charles River Laboratories, Wilmington, MA) by injecting 1,500 iL3 in phosphate buffered saline subcutaneously under the same IACUC protocols; we induced autoinfection by subcutaneously injecting 2 mg methylprednisolone acetate weekly starting with the day of infection^31^. We sacrificed *M. unguiculatus* after 16–18 days by CO_2_ asphyxiation in accordance with standards established by the American Veterinary Medical Association. We recovered the luminal contents of the small and large intestines by suspending the gut segments, split length-wise, in graduated cylinders with Dulbecco's Modified Eagle's Medium (DMEM), supplemented with gentamycin [100 μg/ml], for three hours at 37 °C, as previously described^32^. We performed all protocols and routine care of the animals in strict accordance with both the Guide for the Care and Use of Laboratory Animals of the National Institutes of Health and the ARRIVE guidelines^33^.

### Isolation of *S. stercoralis* free-living adult males for RNA-Seq

To first isolate *S. stercoralis* free-living adults, we incubated charcoal coprocultures^34^ (composed of fresh dog feces and bone charcoal) for 48 h at 21 °C and then used the Baermann technique, with water at 28–29 °C, to induce free-living adults to migrate from the cultures. We then removed a 30 ml aliquot of the Baermann effluent and allowed worms to sediment at 1 × *g* for 10 min. In order to separate the worms from most contaminating bacteria, we mixed ~ 1–2 ml of worms and water with an equal volume of 1% SeaKem low-melting temperature agarose at 30 °C (Lonza, Basel, Switzerland) and transferred this mixture to a petri dish. We then cooled the suspension on a glass slab at 4 °C until the agarose began to gel and then transferred it to room temperature for 10–15 min. We induced the mixture of free-living adult males, free-living adult females, and larvae to migrate from the agarose, which retained most contaminants, by adding 14 ml of BU Buffer^35^ and incubating at 28 °C for 30 min. We then manually isolated and transferred several hundred young free-living adult males to a 1.5 ml microfuge tube with a pipette and allowed them to settle at 1 × *g* for ~ 10 min. We then removed the supernatant and mixed the ~ 10 μl of adult males with 200 μl TRIzol reagent (Thermo Fisher Scientific, Waltham, MA) and snap froze the mixture in liquid nitrogen. This isolation of free-living adult male worms was performed in biological triplicate on different days.

### RNA extraction, library preparation, and sequencing

We extracted total RNA from each replicate of free-living adult males using TRIzol and the manufacturer’s protocol, and we then quantified RNA concentration and determined the RNA integrity number (RIN) using a Bioanalyzer 2100 (Agilent Technologies, Santa Clara, CA). All three samples had a RIN of 10.0. We then constructed libraries, each with unique indexes, using the TruSeq RNA Sample Preparation Kit (Illumina, San Diego, CA) using 500 ng of total RNA as starting material and a previously described protocol^32^. We determined the concentration of the three libraries using the Kapa SYBR Fast qPCR Kit for Library Quantification (Kapa Biosystems, Inc.) and pooled the three libraries as previously described. We then sequenced the three pooled libraries on an Illumina HiSeq 2000 with 100 base pair paired-end reads, and we then demultiplexed the reads using the unique indexes. Raw RNA-Seq reads for each of the three biological replicates of free-living adult males are available in the National Center for Biotechnology Information (NCBI) Sequence Read Archive (SRA) database under BioProject ID: PRJNA689252.

### RNA-Seq read processing and mapping

In addition to raw RNA-Seq reads from free-living adult males, we included raw RNA-Seq reads from *S. stercoralis* free-living adult females, post-free-living L1, iL3, L3 activated inside a permissive host (L3 +), parasitic adult females, post-parasitic L1 (primarily developing heterogonically), and post-parasitic L3 enriched for females (also developing heterogonically) in our analysis from previously described data^32^, available under BioProject ID: PRJEB3116. Since these libraries were constructed at the same time as those derived from free-living adult males and were sequenced using the same chemistry and instrument, but at an earlier date, batch effects should be minimized. To assess read quality, we used FastQC v.0.11.9^36^. We then trimmed low-quality bases and index sequences with Trimmomatic v.0.39^37^ and the options ILLUMINACLIP:2:30:10:1 SLIDINGWINDOW:2:10 LEADING:10 TRAILING:10 MINLEN:40. We confirmed the removal of the low-quality bases and index sequences with FastQC. We subsequently removed contaminating rRNA sequences from the trimmed reads using bbduk.sh from bbtools v.38.84^38^ and a list of *S. stercoralis* 18S, 5.8S, 28S, and 5S rRNA sequences, including accession numbers M84229, AF279916, KU180693, DQ145710, EF653265, and sequences from the PV001 strain derived from previously de novo assembled RNA-Seq reads^32^. We mapped the processed reads to the *S. stercoralis* genome v2.0.4^39^ using HISAT2 v.2.2.0^40^ with the max intron length set to 50,000. We then converted the output SAM files to BAM files with SAMtools v.1.9^41^.

### Identification of *S. stercoralis* homologs

We identified *S. stercoralis* homologs of canonical *C. elegans* sex-determination genes by reciprocal Basic Local Alignment Search Tool (BLAST) searches. We first used *C. elegans* polypeptide sequences as queries for BLAST searches against the *S. stercoralis* genome^39^ using Geneious v.11.1.5 (Biomatters, Ltd., Auckland, New Zealand). We then corrected or confirmed the annotation for each *S. stercoralis* gene using RNA-Seq reads aligned to the genome and visualized using the Integrative Genome Viewer v.2.3.94^42^. We then predicted protein sequences of *S. stercoralis* homologs using the annotated transcripts in Geneious and subsequently used the predicted protein sequences as protein BLAST queries against the *C. elegans* database in NCBI to confirm gene identity. Only genes with top hits in reciprocal BLAST searches, where the second hit had a significantly higher e-value in both searches or was resolved by phylogenetic analysis (see below), were identified as one-to-one homologs. We manually adjusted any changes in the *S. stercoralis* genome annotations (Supplemental Data S1). All annotated *S. stercoralis* transcripts include the prefix *Ss*.

We used *C. elegans* and *S. stercoralis* predicted protein sequences as NCBI protein BLAST search queries to identify related predicted polypeptides in clade I, III, IV, and V nematodes^28^. We additionally performed BLAST searches of parasitic nematode genomes available on WormBase ParaSite^43^. We aligned the protein sequences with ClustalW and a BLOSUM matrix and then created neighbor-joining (N-J) phylogenetic trees with 100 iterations of bootstrapping in Geneious. Accession numbers or gene IDs for additional sequences are listed in the respective phylogenetic trees.

To determine whether predicted polypeptide sequences had a predicted signal peptide, we utilized SignalP v.5.0 server^44^. Similarly, to determine whether a predicted polypeptide had predicted transmembrane domains, we utilized TMHMM Server v.2.0^45^.

### Transcript abundance quantification and differential expression analysis

To quantify transcript abundance for each gene, we first converted the updated GFF3 annotation file to a GTF file using custom scripts and gffread v.0.11.7^46^ and then quantified the paired-end fragment counts for the coding sequence (CDS) of each gene using featureCounts from SubReads v.2.0.1^47^. We performed trimmed mean of M-values (TMM) normalization of counts per million (CPM) values for each gene in each biological replicate (Supplemental Data S2), using featureCounts summary data and EdgeR v.3.32.0^48,49^. We performed differential gene expression analysis between developmental stages with glmQLFit and glmTreat functions in the EdgeR package using a significance threshold of: a minimum log_2_ fold change of one, a minimum mean log_2_ CPM of one in at least one developmental stage, a p-value adjusted threshold of < 0.05, and a p-value adjustment using Benjamini–Hochberg false-discovery-rate (FDR) (Supplemental Data S3).

### Data visualization

Rhabditiform males recovered from experimentally infected Mongolian gerbils were wet-mounted without anesthetics using an Olympus BX60 compound microscope equipped with differential interference contrast (DIC) optics, a Spot RT Color digital camera, and Spot Advanced v5.1 image analysis software (Diagnostic Instruments, Inc., Sterling Heights, MI).

We used EdgeR to generate a multidimensional scaling (MDS) plot of all samples and a mean-difference (MD) plot of differential gene expression between free-living adult males and free-living adult females. We plotted the TMM-normalized CPM for each replicate, the mean TMM-normalized CPM, and error bars that represent 95% confidence intervals for the three replicates of each developmental stage in GraphPad Prism v.9.0.0 (GraphPad Software, Inc., San Diego, USA).

## Results and discussion

### S. stercoralis males are invariably rhabditiform

*S. stercoralis*, along with other *Strongyloides* species, has a unique life cycle with two types of adults: a parasitic form that is a parthenogenetic female, while the free-living forms are gonochoristic and reproduce sexually. Although Kreis (1932) detailed morphological descriptions of *S. stercoralis* parasitic males^7^, Faust (1933) described parasitic males in the ileum and rectum of the host^8^, and others have found rhabditiform males in the lungs^50^, it is now generally accepted that the parasitic generation is only female^3^. Interestingly, we have observed *S. stercoralis* L4/adult rhabditiform males, with characteristic enlarged pharyngeal bulbs and pair of copulatory spicules, in the large intestine of experimentally infected Mongolian gerbils that have been treated with corticosteroids and are undergoing hyperinfection (Supplemental Figure S1). These males are morphologically similar to those described by Kreis^7^.

We hypothesize that these rhabditiform males are precociously developing free-living males—an observation consistent with the male sex of the post-parasitic generation of *S. stercoralis* being determined by the inheritance of a single X chromosome, such that genetically male post-parasitic larvae invariably develop towards a rhabditiform free-living larva whether they are in the environment or in the host^6^. Furthermore, our findings suggest that corticosteroid treatment not only triggers precocious development of post-parasitic female larvae, but also post-parasitic male larvae. While the mechanism by which corticosteroid treatment triggers autoinfection is unknown, our findings suggest that corticosteroids may directly influence development of post-parasitic larvae. Whether precociously developing rhabditiform males up-regulate astacin-like metallopeptidases, which are up-regulated in iL3^39,51^ and important for skin penetration^52^, or whether they are capable of penetrating the gut wall and migrating within the host also remains unknown.

### The transcriptional profile of S. stercoralis free-living adult males is distinct from other developmental stages

Since little is known about the changes in gene expression that control development of post-parasitic larvae fated for free-living male or female development, we examined changes in transcript abundance between free-living adult males and free-living adult females using RNA-Seq. We isolated *S. stercoralis* free-living adult males in biological triplicate and used the three amassed cohorts to construct RNA-Seq libraries. We sequenced the libraries on an Illumina HiSeq 2000, generating a mean of 46.2 million paired-end reads (± 1.4 million, standard error of the mean) per sample.

**Figure 2:**
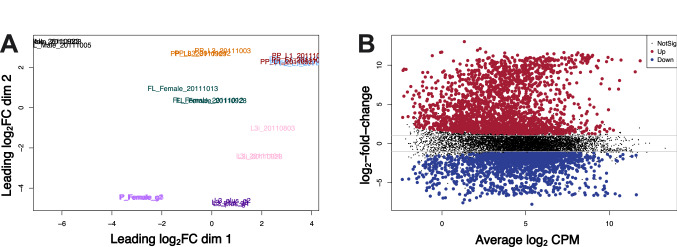
*S. stercoralis* developmental stages have distinct transcript expression profiles. (**A**) *S. stercoralis* developmental stages examined by RNA-Seq are well-separated, and the three biological replicates group closely, by multidimensional scaling (MDS) analysis. (**B**) Numerous transcripts are significantly (log_2_ fold change > 1.0; FDR < 0.05) up-regulated (red) and down-regulated (blue) in free-living adult males, in comparison to free-living adult females, by mean-difference (MD) analysis. A subset of transcripts are significantly up-regulated in free-living adult males to a greater extent than transcripts are down-regulated. Plots were constructed using EdgeR v.3.32.0.

To identify differences in transcript abundance between *S. stercoralis* free-living adult males and other developmental stages^32^, we characterized each developmental stage in biological triplicate using RNA-Seq followed by TMM normalization and statistical testing using EdgeR. An MDS plot that arranged each sample in space based on similarities/differences in transcript abundance revealed that biological replicates grouped tightly together, while developmental stages were well separated (Fig. 2A). An MD plot comparing differences in transcript abundance between free-living adult males and free-living adult females revealed 2,215 transcripts that were significantly up-regulated in free-living adult males and 2,046 transcripts that were significantly up-regulated in free-living adult females (Fig. 2B). We also observed a subset of genes that were up-regulated to a greater extent in free-living adult males than genes up-regulated in free-living adult females (Fig. 2B). A similar observation has been made in males of several *Caenorhabditis* species^53^.

### Transcripts encoding major sperm proteins and two clusters of predicted transmembrane polypeptides are among the most highly up-regulated transcripts in free-living adult males

To investigate which transcripts are most up-regulated in *S. stercoralis* free-living adult males in comparison to free-living adult females, we characterized the top 15 most up-regulated transcripts in free-living adult males (Table 1). Three of these transcripts are predicted to encode canonical 127 amino acid major sperm proteins (MSPs), which are conserved nematode-specific proteins that form stable dimers and assemble into actin-like filaments that are crucial for sperm motility^54^. In contrast to the *C. elegans* genome, which has 31 MSP-encoding genes^55^, we were only able to identify three canonical 127 amino acid MSP-encoding genes in the *S. stercoralis* genome.

**Table 1:** Most up-regulated transcripts in *S. stercoralis* free-living adult males in comparison to free-living adult females.

Gene name	*Log_2_ fold change	Predicted number of amino acids	Signal peptide likelihood	Predicted transmembrane domain(s)	Predicted homology and/or function
*SSTP_0001266600*	13.0	126	0.7582	102–124 aa	*C. elegans*—no significant similarity *S. ratti*—similar proteins, but no 1-to-1 ortholog
*SSTP_0000642100*	12.1	208	0.9393	–	*C. elegans*—no significant similarity *S. ratti*—similar proteins, but no 1-to-1 ortholog
*SSTP_0000017020*	11.9	59	0.0005	18–40 aa	*C. elegans*—no significant similarity *S. ratti*—no significant similarity
*SSTP_0000031000*	11.7	79	0.9996	–	*C. elegans*—no significant similarity *S. ratti*—hypothetical protein XP_024503864.1 (e-11)
*SSTP_0001268100*	11.6	182	0.9876	158–180 aa	*C. elegans*—no significant similarity *S. ratti*—similar proteins, but no 1-to-1 ortholog
*SSTP_0001268000*	11.6	219	0.0041	125–147 aa	*C. elegans*—no significant similarity *S. ratti*—similar proteins, but no 1-to-1 ortholog
*SSTP_0000894400*	11.6	223	0.9989	183–205 aa	emp24/gp25L/p24/GOLD family protein, which are involved in vesicular protein trafficking from the ER
*SSTP_0000040300*	11.6	90	0.0011	–	*C. elegans*—no significant similarity *S. ratti*—hypothetical protein XP_024505868.1 (e-23)
*SSTP_0000384900*	11.5	129	0.9783	–	*C. elegans*—no significant similarity *S. ratti*—no significant similarity
*Ss_msp_3*	11.4	127	0.0006	–	major sperm protein (canonical)
*SSTP_0001267400*	11.4	190	0.9612	95–117 aa	*C. elegans*—no significant similarity *S. ratti*—similar proteins, but no 1-to-1 ortholog
*Ss_msp_1*	11.4	127	0.0007	–	major sperm protein (canonical)
*SSTP_0001267900*	11.4	189	0.9966	168–187 aa	*C. elegans*—no significant similarity *S. ratti*—similar proteins, but no 1-to-1 ortholog
*Ss_msp_2*	11.3	127	0.0008	–	major sperm protein (canonical)
*SSTP_0000016700*	11.3	110	0.0014	10–32 aa	*C. elegans*—no significant similarity *S. ratti*—no significant similarity

*For significance: FDR < 0.05, log_2_ fold change > 1.0, and log_2_ CPM > 1.0 in ≥ 1 developmental stage.

Intriguingly, we noted that several of the most up-regulated genes were clustered in the genome (Table 1). Upon further investigation, we identified two gene clusters, one on scaffold 5 and one on scaffold 9, where each gene in the cluster is highly expressed only in free-living adult males (Supplemental Figures S2 and S3). The cluster on scaffold 5 includes 12 genes, with the majority encoding predicted polypeptides that lack a signal peptide and are predicted Type I single-pass transmembrane proteins (N-term is extracellular and C-term is cytoplasmic) with the transmembrane domain located near the middle of the predicted polypeptide (Supplemental Table S1). None of these 12 predicted polypeptides have BLAST hits in *S. ratti* or *C. elegans*, nor do they have any clear phylogenetic relationships with each other. The cluster on scaffold 9 includes 14 genes and one pseudo-gene, with the majority encoding predicted polypeptides with both a predicted signal peptide and a predicted Type I single-pass transmembrane domain, with the transmembrane domain located near the C-terminus (Supplemental Table S2). Although we were able to identify seven homologs of these genes in *S. ratti* as well as one homolog in *P. trichosuri*, we were unable to identify any one-to-one homologs or clear phylogenetic relationships.

Our data suggest that the genes in these two clusters encode transmembrane proteins that are specific to *S. stercoralis* and are only present in free-living males. We hypothesize these transmembrane proteins may be important for sperm development or function, as they are up-regulated to a similar extent as transcripts encoding MSPs. Furthermore, these genes appear to be undergoing rapid sequence evolution, as they have little direct homology to each other or to genes outside of *S. stercoralis*. These genes may have evolved under similar selective pressures as the *male secreted short* (*mss*) genes that encode glycosylphosphatidylinositol (GPI)-anchored proteins found in spermatocytes of outcrossing *Caenorhabditis* species and are required for sperm competitiveness^56^.

### Ss-srg-14 encodes a G protein-coupled receptor that is up-regulated in free-living adult males

In *C. elegans*, hermaphrodites produce ascaroside pheromones that attract males^57^, and pheromones have been described in *P. trichosuri*^58^. Since G protein-coupled receptors (GPCRs) play a role in sensing these pheromone cues^59–61^, we sought to determine whether any putative GPCR-encoding transcripts^62^ are up-regulated in *S. stercoralis* free-living males (Supplemental Figure S4). We found that *Ss-srg-14* is highly expressed in males and 8.5-fold up-regulated in free-living adult males in comparison to free-living adult females (FDR < 0.05). We hypothesize that the GPCR encoded by *Ss-srg-14* may be important for the detection of female pheromones. Future studies using CRISPR/Cas9 to knock out *Ss-srg-14* may be informative in determining its biological function.

### TRA-1 and MAB-3, which regulate sex determination in *Caenorhabditis* species, are present in *S. stercoralis* and may have similar functions

A variety of genetic techniques have been used to identify genes that regulate sex determination in *C. elegans*, *C. briggsae*, and other *Caenorhabditis* species^26^ (Fig. 1A); henceforth, *Caenorhabditis* genes do not have prefixes. These genes fall into several broad categories: genes that recognize the X chromosome to autosome ratio (e.g., *sex-1* and *fox-1*) and integrate this signal (*xol-1*), genes that are part of the dosage compensation complex (e.g., *sdc-1*, *sdc-2*, and *sdc-3*), genes involved in a secreted ligand and membrane receptor pathway (e.g., *her-1*, *tra-2*, *tra-3*, *fem-1*, *fem-2*, and *fem-3*), the global regulator *tra-1*, and tissue-specific response genes (e.g., *fog-1*, *fog-3*, and *mab-3*)^26^. In order to identify *S. stercoralis* homologs of these genes, we utilized both BLAST searches and phylogenetic analyses. To determine whether our inability to find some *S. stercoralis* homologs of *Caenorhabditis* sex determination genes was due to an incomplete genome assembly, we performed BLAST searches of other clade III, IV, and V nematode species (Table 2). We then examined changes in transcript abundance of the *S. stercoralis* homologs throughout the life cycle (Fig. 1B-H; Supplemental Figure S5). Although differences in transcript abundance are not necessary for a gene to regulate sex determination, as *Caenorhabditis* species employ multiple post-transcriptional regulatory mechanisms in the sex determination pathway, differences in transcript abundance between free-living adult males and free-living adult females of *S. stercoralis* homologs help to elucidate transcripts that are sex-biased.

**Table 2:** Homologs of genes involved in *Caenorhabditis* sex determination in representative clade III, IV, and V nematodes.

	*xol-1*	*sdc-1*	*sdc-2*	*sdc-3*	*her-1*	*tra-2*	*tra-3*	*fem-1*	*fem-2*	*fem-3*	*tra-1*	*mab-3*
**Clade V**												
*Caenorhabditis elegans*	+	+	+	+	+	+	+	+	+	+	+	+
*Caenorhabditis briggsae*	+	+	+	+	+	+	+	+	+	+	+	+
*Pristionchus pacificus*	−	+/−*	−	−	+	−	+	+	+	−	+	+
*Ancylostoma ceylancum*	−	+/−*	+	−	+	−	+	+	+	+	+	+
*Haemonchus contortus*	−	+/−*	+	−	+	−	+	+	+	+	+	+
**Clade IV**												
*Strongyloides stercoralis*	−	+/−*	−	−	+	−	+	−	+	−	+	+
*Strongyloides ratti*	−	+/−*	−	−	−	−	+	−	+	−	+	+
*Strongyloides papillosus*	−	+/−*	−	−	+	−	+	−	+	−	+	+
*Bursaphelenchus xylophilus*	−	+/−*	−	−	−	−	+	−	+	−	+	+
*Meloidogyne enterolobii*	−	+/−*	-	−	−	−	+	−	+	−	+	+
**Clade III**												
*Ascaris suum*	−	+/−*	−	−	−	−	+	+	+	−	+	+
*Brugia malayi*	−	+/−*	−	−	+	−	+	+	+	−	+	+
*Loa loa*	−	+/−*	−	−	+	−	+	+	+	−	+	+
*Toxocara canis*	−	+/−*	−	−	+	−	+	+	+	−	+	+

*Unclear whether these homologs are more similar to *Caenorhabditis elegans sdc−1* or *C04F5.9*.

We identified *S. stercoralis* homologs of several “core” sex determination genes (Fig. 1A), including *tra-1*, which encodes a zinc-finger transcription factor related to *Drosophila* cubitus interruptus (Ci) that also plays a role in *P. pacificus* sex determination^63,64^, and *mab-3*, which encodes a doublesex (Dsx) -related transcription factor^65^. We found that the *S. stercoralis* homolog of the masculinizing *mab-3* gene, *Ss-mab-3*, is 7.9-fold up-regulated in free-living adult males in comparison to free-living adult females (FDR < 0.05), which is consistent with *mab-3* up-regulation in *C. elegans* males^66^. Furthermore, we identified homologs of genes encoding TRA-1 and MAB-3 in all clade III, IV, and V nematodes that we examined (Table 2). Due to the broad conservation of these genes across nematode species and their role in sex determination in other ecdysozoans, we hypothesize that *tra-1* and *mab-3* play a similar role in sex determination in *S. stercoralis* and other nematodes.

In *Caenorhabditis* species, TRA-1 activity is repressed in males by a FEM-1, FEM-2, FEM-3, and CUL-2 complex that ubiquitinates TRA-1 and targets it for degradation via the proteasome^67^. We identified *S. stercoralis* homologs of the genes *fem-2*, which encodes a PP2C phosphatase^68^, *sel-10*, which encodes an F-box protein that is a component of E3 ubiquitin ligases^69^, and *cul-2*, which encodes a cullin family protein that functions in ubiquitin ligase complexes^67^. *S. stercoralis* homologs of *fem-2* and *sel-10* are significantly up-regulated in free-living adult females in comparison to free-living adult males (5.1-fold and 3.0-fold up-regulated for *Ss-fem-2* and *Ss-sel-10*, respectively; FDR < 0.05). Whether *Ss-fem-2* and *Ss-sel-10*, which are members of gene families with functions outside of sex determination^70^, have a conserved role in *S. stercoralis* sex determination will require future functional studies.

We were only able to identify homologs of genes encoding FEM-3, which directly interacts with the C-terminal domain of TRA-2 in *Caenorhabditis* species^71^, in a few clade V nematodes (Table 2). As *fem-3* appears to be rapidly evolving and has significant sequence divergence between *Caenorhabditis* species^72^, we may be unable to detect divergent homologs in clade III and IV nematodes by sequence homology. Interestingly, we were unable to identify homologs of genes encoding FEM-1, which is an E3 ubiquitin ligase subunit that recognizes substrates—including TRA-1—for targeted ubiquitination^67^, in clade IV parasites, but we were able to identify homologs in clade III and V nematodes (Table 2). Since *Ss-tra-1* transcript abundance does not differ significantly between free-living adult males and females (Fig. 1F), we hypothesize that the *S. stercoralis* homolog of TRA-1 is still regulated post-translationally. However, the apparent loss of *fem-1* in both *S. stercoralis* and other clade IV nematodes suggests that these parasites may have a modified mechanism of regulating TRA-1 activity.

### The HER-1 ligand and TRA-2 receptor, which regulate sex determination in *Caenorhabditis* species, appear to have been lost in some parasitic nematode lineages

In *C. elegans*, the HER-1 ligand is expressed in males and represses the TRA-2A transmembrane receptor^73^, resulting in the repression of FEM-3^71^. Additionally, TRA-3 can cleave the C-terminus of TRA-2A^74^, with the resulting fragment having feminizing activity via binding to FEM-3^71^. We were unable to identify an *S. stercoralis* homolog of *tra-2*, which in *C. elegans* encodes a 1475 amino acid 12 transmembrane protein^75^ that is directly bound by the ligand encoded by *her-1*^73^. Although we were able to identify conserved HER-1 homologs in *S. stercoralis* and several other clade III^76^, IV, and V nematodes (Supplemental Figure S6; Table 2), we were unable to identify TRA-2 homologs outside of the *Caenorhabditis* genus—despite the ready identification of the related Patched-1 and Patched-3 homologs^75^ in clade III, IV, and V nematodes (Supplemental Figure S7; Table 2). Intriguingly, *Ss-her-1* was not expressed in any of the *S. stercoralis* developmental stages we examined, and the remnant of the *S. ratti her-1* coding sequence, located between *SRAE_2000507500* and *SRAE_2000507600*, has acquired several stop codons and deletions. In addition to *S. ratti*, we were unable to identify genes encoding HER-1 homologs in several disparate species (Table 2). By contrast, genes encoding TRA-3, a calpain protease that cleaves TRA-2A^74^, were broadly conserved among the parasitic nematode species we examined (Table 2).

Our observation that TRA-2 homologs are not readily identifiable by BLAST searches outside of the *Caenorhabditis* genus suggests that either TRA-2 has evolved so rapidly that it is not distinguishable by protein sequence homology in other species or that another protein functions as the receptor for HER-1 in other nematode species. We could not identify a TRA-2 homolog in either *Diploscapter coronatus* or *D. pachys*, but we were able to identify a TRA-2A homolog in *C. parvicauda* (14.5% identity to *C. elegans* TRA-2A), which is the most distantly-related *Caenorhabditis* species from *C. elegans* with a sequenced genome^77^. By contrast, the HER-1 sequence is conserved across the clade III, IV, and V nematode species in which it is present. Therefore, we speculate that TRA-2 may be a rapidly-evolving Patched-1/-3 paralog in *Caenorhabditis* species. Alternatively, homologs of the several hedgehog-like ligands in *C. elegans*^78^, of which HER-1 is a member, could function in sex determination in other nematode species. This is supported by the loss of *her-1* in parasites such as *S. ratti*. Finally, it is also possible that HER-1 and TRA-2 have simply been co-opted into a role in sex determination in *Caenorhabditis* species.

### Proteins that respond to the X chromosome to autosome ratio signal in *Caenorhabditis* species appear to be rapidly evolving

In *Caenorhabditis* species, XOL-1 is active only in males and integrates the X chromosome to autosome ratio signal, which is crucial for dosage compensation and down-regulation of gene expression from X chromosomes to male levels in XX worms^79^. Activated *xol-1* represses the function of *sdc-1*, *sdc-2*, and *sdc-3* (formerly *dpy-29*), which then permits *her-1* expression^80,81^. We were unable to identify homologs of genes encoding either XOL-1, which is structurally related to GHMP small molecule kinases^82^, or SDC-3, which has zinc-finger motifs^83^, outside of *Caenorhabditis* species (Table 2). Similarly, we were only able to identify weak homologs of genes encoding SDC-2, which directly represses *her-1* expression in *C. elegans*^80^, in a few clade V parasites (Table 2). Identifiable *xol-1*, *sdc-2*, and *sdc-3* homologs also appear to be absent from the clade III parasitic nematode *Brugia malayi*^15^. We were unable to clearly identify an *S. stercoralis* homolog of *sdc-1*, which encodes a zinc-finger transcription factor^84^, because the top BLAST hit, *SSTP_0000950300*, encodes a protein that is equally related to SDC-1 and C04F5.9 in *C. elegans* and other *Caenorhabditis* species. Since *xol-1* appears to be rapidly evolving and has significant sequence divergence between *Caenorhabditis* species^82^, and *sdc-2* homologs appear to have similar sequence divergence, we may simply be unable to detect homologs of these genes in clade III and IV nematodes by sequence homology. Alternatively, a true loss of *xol-1*, *sdc-2*, and/or *sdc-3* homologs in *S. stercoralis* may suggest a different mechanism of regulating dosage compensation of the X chromosome between XX and XO individuals. Whether *S. stercoralis* actually balances gene expression on the X chromosome between males and females is unknown, although sex-specific histone H3 modifications, which play a role in dosage compensation in other species, have been observed in the *S. ratti* male germline^23^.

### Transcriptomic profiles of nuclear hormone receptors and cytochrome P450s are associated with sex

**Figure 3:**
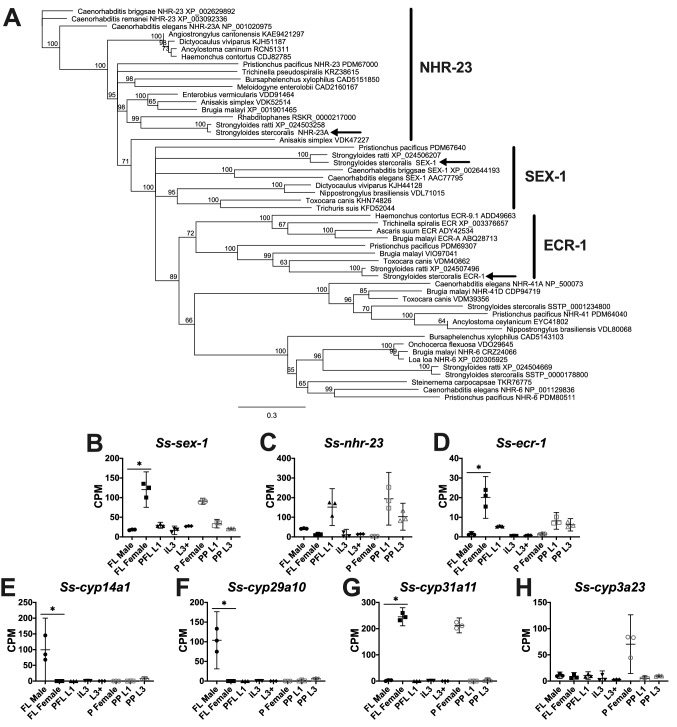
Transcripts encoding *S. stercoralis* nuclear hormone receptors and cytochrome P450s are differentially regulated between males and females. (**A**) Homologs of SEX-1, NHR-23, and ECR-1 in *S. stercoralis* (arrows) and other clade I, III, IV, and V nematodes are identified in a neighbor-joining phylogenetic tree with 100 iterations of bootstrapping constructed with Geneious v.11.1.5. Gene names and accession numbers are listed after the species names. (**B**-**H**) Transcript abundances of the *S. stercoralis* homologs encoding relevant nuclear hormone receptors and cytochrome P450s are represented using TMM-normalized counts per million (CPM) for the following developmental stages: free-living adult males (FL Male), free-living gravid adult females (FL Female), post-free-living first-stage larvae (PFL L1), developmentally arrested infectious third-stage larvae (iL3), L3 activated inside a permissive host (L3 +), parasitic gravid adult females (P Female), heterogonically-developing post-parasitic L1 (PP L1), and heterogonically-developing post-parasitic L3 enriched for females (PP L3). Graphs were constructed using GraphPad Prism v.9.0.0; bars indicate means (horizontal) and 95% confidence intervals (vertical) for each of the three biological replicates represented as individual data points. Asterisks (*) indicate a significant (fold change > 2.0; FDR < 0.05) difference between FL Male and FL Female.

The *C. elegans* genome is predicted to encode 284 nuclear hormone receptors^85^, including SEX-1 and NHR-23, which function in sex determination and spermatogenesis, respectively, among other processes^86,87^. While both SEX-1 and NHR-23 are related to the ecdysone nuclear hormone receptor ECR-1, *Caenorhabditis* species do not have ECR-1 homologs, in contrast to most other nematode species^88^. Therefore, we sought to identify *S. stercoralis* homologs of *sex-1*, *nhr-23*, and *ecr-1*, and determine whether their transcripts were regulated in a sex-specific manner. Using reciprocal BLAST searches, we identified *S. stercoralis* homologs of these three putative nuclear hormone receptors (Fig. 3A). We found that both *Ss-sex-1* and *Ss-ecr-1* transcripts are significantly up-regulated in free-living adult females in comparison to free-living adult males (6.6-fold and 14.2-fold, respectively; FDR < 0.05), while transcripts for *Ss-nhr-23* are up-regulated in developing larvae—consistent with its role in regulating molting in *C. elegans*^89^ (Fig. 3B-D).

While the native ligand for ECR-1 in parasitic nematodes is likely 20-hydroxyecdysone^90^, the endogenous ligands for SEX-1 and NHR-23 have not yet been described, but are hypothesized to be steroid hormones^91^. Therefore, we asked whether any of the 26 *S. stercoralis* cytochrome P450-encoding transcripts^14^ are regulated in a sex-specific manner and thus might produce a sex-specific hormone. We found that *Ss-cyp14a1* and *Ss-cyp29a10* transcripts were significantly up-regulated in free-living adult males in comparison to free-living adult females (565-fold and 832-fold, respectively; FDR < 0.05) (Fig. 3E-F). Consequently, we hypothesize these two genes may be responsible for the production of androgens. We also found that, of the developmental stages we examined, *Ss-cyp31a11* transcripts were solely found in adult free-living and parasitic females and *Ss-cyp3a23* transcripts were only found in parasitic females (Fig. 3G-H). We hypothesize that *Ss-cyp-31a11* may encode the cytochrome P450 necessary for ecdysone biosynthesis in *S. stercoralis*, as its regulation through *S. stercoralis* development is consistent with that of *Ss-ecr-1*. Alternatively, these sex-specific cytochrome P450s may be involved in the biosynthesis of sex-specific pheromones or other signaling molecules.

### Transcripts encoding putative mRNA-binding proteins are sex specific

**Figure 4:**
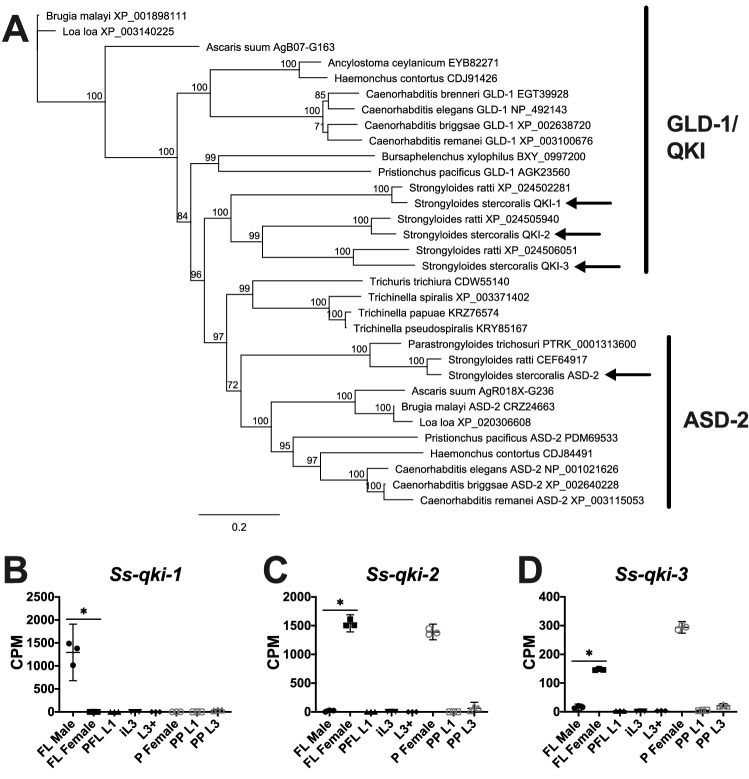
The *S. stercoralis* genome encodes three GLD-1 paralogs with transcripts that are sex specific. (**A**) Homologs of GLD-1 and ASD-2 in *S. stercoralis* (arrows) and other III, IV, and V nematodes are identified in a neighbor-joining phylogenetic tree with 100 iterations of bootstrapping constructed with Geneious v.11.1.5. Gene names and accession numbers are listed after the species names. (**B-D**) Transcript abundances of the three *S. stercoralis* paralogs are represented using TMM-normalized counts per million (CPM) for the following developmental stages: free-living adult males (FL Male), free-living gravid adult females (FL Female), post-free-living first-stage larvae (PFL L1), developmentally arrested infectious third-stage larvae (iL3), L3 activated inside a permissive host (L3 +), parasitic gravid adult females (P Female), heterogonically-developing post-parasitic L1 (PP L1), and heterogonically-developing post-parasitic L3 enriched for females (PP L3). Graphs were constructed using GraphPad Prism v.9.0.0; bars indicate means (horizontal) and 95% confidence intervals (vertical) for each of the three biological replicates represented as individual data points. Asterisks (*) indicate a significant (fold change > 2.0; FDR < 0.05) difference between FL Male and FL Female.

The STAR domain protein GLD-1 represses translation via mRNA 3′ UTR binding and regulates the transition of mitosis to meiosis and development of germ cells in *Caenorhabditis* species^92^, although specific molecules bound by *C. elegans* and *C. briggsae* GLD-1 homologs vary^93^. To identify a *gld-1* homolog in *S. stercoralis* and differentiate it from the closely-related *asd-2*, we performed reciprocal BLAST searches and constructed a phylogenetic tree. We identified three *gld-1* paralogs in the *S. stercoralis* genome (Fig. 4A), which we termed *Ss-qki-1*, *Ss-qki-2*, and *Ss-qki-3*. Strikingly, of the developmental stages we examined, *Ss-qki-1* transcripts are only expressed in free-living adult males (~ 700-fold up-regulated in comparison to free-living adult females; FDR < 0.05), while *Ss-qki-2* and *Ss-qki-3* transcripts are specifically and highly expressed in both free-living and parasitic adult females (Fig. 4B-D). We hypothesize that an ancestral *gld-1* homolog was duplicated twice, resulting in three paralogs in *Strongyloides* species, with *Ss-qki-1* taking on adult male-specific functions and *Ss-qki-2* and *Ss-qki-3* taking on adult female-specific functions. Future studies to identify the molecular targets of the proteins these genes encode may aid in determining their functions.

**Figure 5:**
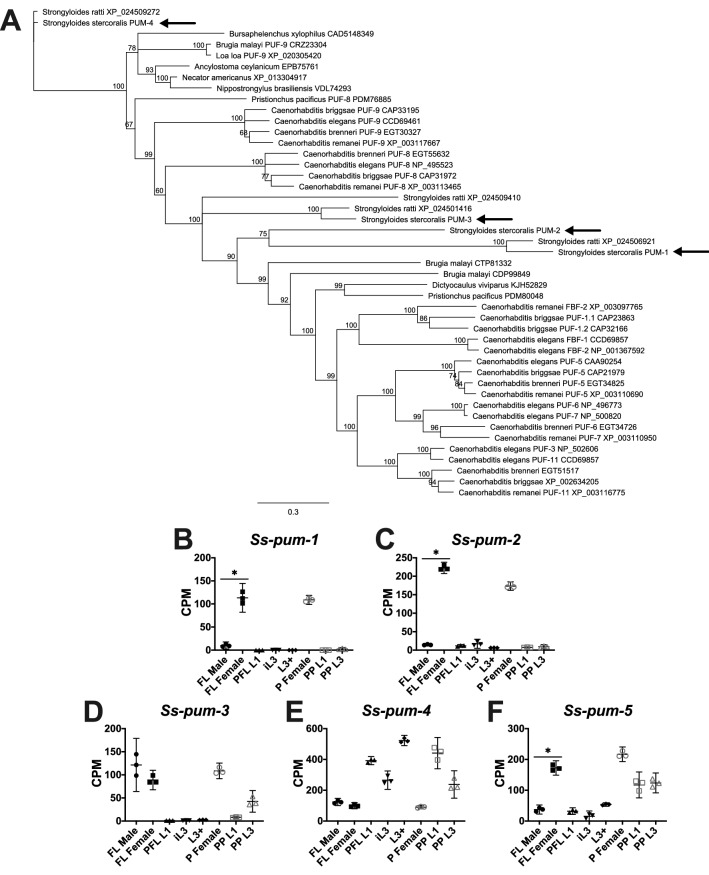
Genes encoding pumilio family proteins appear to have been duplicated in *Strongyloides* species. (**A**) Homologs of pumilio family proteins in *S. stercoralis* (arrows) and other clade III, IV, and V nematodes are identified in a neighbor-joining phylogenetic tree with 100 iterations of bootstrapping constructed with Geneious v.11.1.5. The genes encoding *Ss*-PUM-1, *Ss*-PUM-2, and *Ss*-PUM-3 appear to have resulted from a duplication in an ancestor of *Strongyloides* species. Gene names and accession numbers are listed after the species names. (**B**-**F**) Transcript abundances of the *S. stercoralis* homologs are represented using TMM-normalized counts per million (CPM) for the following developmental stages: free-living adult males (FL Male), free-living gravid adult females (FL Female), post-free-living first-stage larvae (PFL L1), developmentally arrested infectious third-stage larvae (iL3), L3 activated inside a permissive host (L3 +), parasitic gravid adult females (P Female), heterogonically-developing post-parasitic L1 (PP L1), and heterogonically-developing post-parasitic L3 enriched for females (PP L3). Graphs were constructed using GraphPad Prism v.9.0.0; bars indicate means (horizontal) and 95% confidence intervals (vertical) for each of the three biological replicates represented as individual data points. Asterisks (*) indicate a significant (fold change > 2.0; FDR < 0.05) difference between FL Male and FL Female.

In *Caenorhabditis* species, the *gld-1* mRNA is regulated by pumilio domain-containing mRNA-binding proteins^94^, which additionally regulate sex determination by binding to other mRNA targets^26^. We sought to identify *S. stercoralis* genes encoding pumilio family proteins by reciprocal BLAST searches and phylogenetic analyses. We found that the genes encoding pumilio family proteins appear to have been duplicated, not only in *Caenorhabditis* species^95^, but also in the common ancestor of *Strongyloides* species (Fig. 5A). The predicted polypeptides encoded by *Ss-pum-1*, *Ss-pum-2*, and *Ss-pum-3* are more similar to each other than to other nematode pumilio family proteins, suggesting they are paralogs (Fig. 5A). *Ss-pum-5* encodes a predicted polypeptide that is homologous to *C. elegans* PUF-12; these homologs are highly divergent from the other pumilio family proteins found in nematode species (data not shown). *Ss-pum-1* and *Ss-pum-2* transcripts are abundant in both free-living and parasitic adult females, but depleted in the other developmental stages we examined (Fig. 5B-C). *Ss-pum-3*, *Ss-pum-4*, and *Ss-pum-5* transcripts have more complex patterns of developmental regulation (Fig. 5D-E), although *Ss-pum-5* transcript abundance (Fig. 5F) is significantly lower in free-living adult males than free-living adult females (4.6-fold up-regulated in free-living adult females in comparison to free-living adult males; FDR < 0.05). We hypothesize that the proteins encoded by *Ss-pum-1* and *Ss-pum-2* play an important role in oogenesis or adult female development.

## Conclusions

Our studies in *S. stercoralis* have revived the idea that post-parasitic males can precociously develop inside the host; whether these rhabditiform males are capable of tissue penetration warrants further study. Additionally, we have identified several *S. stercoralis* sex-specific transcripts that encode putative male-specific single-pass transmembrane domain proteins, cytochrome P450s, and a GPCR that may be involved in sex-specific functions. We have also identified *S. stercoralis* homologs of multiple genes that regulate sex determination in *Caenorhabditis* species as well as several specific differences, including three paralogs of *gld-1* (*Ss-qki-1*, *Ss-qki-2*, and *Ss-qki-3*) and divergent homologs of pumilio domain-containing proteins (*Ss-pum-1*, *Ss-pum-2*, and *Ss-pum-3*). Several of these genes also have sex-specific transcripts. Although the molecular mechanisms controlling sex determination and male development in *S. stercoralis* remain far from resolved, our studies have identified several genes that warrant further study. Utilization of established techniques for transgenesis and targeted mutagenesis in *S. stercoralis* could help elucidate the biological functions of these genes in the parasite^5^.

More broadly, nematodes utilize a variety of mechanisms for sex determination. Although most nematodes use an XX/XO sex chromosome system, XX/XY systems also exist^15^, and the original signals that determine the combination of sex chromosomes in a developing embryo can be strictly genetic or environmentally-influenced^19^. Additionally, there are a few nematode species without autonomous X chromosomes; in *S. papillosus* and *S. vituli*, sex-specific chromatin diminution is necessary for male development^19^. In *Caenorhabditis* species, where sex is genetically determined, the genes responsible for sensing the X chromosome to autosome ratio, propagating this signal, and executing somatic and germline programs in the different sexes have been extensively studied^26^. Although core components are conserved between *C. elegans* and other *Caenorhabditis* species as well as *P. pacificus*, there are several differences^26^. These observations have led to the “bottom up” hypothesis, which posits that the most down-stream components are conserved and that up-stream components have evolved sequentially^27^. It has also been hypothesized that the genes from *her-1* to *tra-1* are derived from the *hedgehog* pathway and compose an indivisible “cassette”^96^.

Our data generally support the “bottom up” hypothesis, with the conservation of *tra-1* and *mab-3* across parasitic nematode species and less conservation (or at least more divergence in sequence) of more upstream members (e.g., *xol-1*, *sdc-2*, and *sdc-3*). However, the apparent loss of *her-1* in several lineages, absence of *fem-1* homologs in clade IV nematodes, and the potential absence of *tra-2* outside of *Caenorhabditis* species, suggest that the “cassette” of genes from *her-1* to *tra-1* may not be indivisible. Even when genes are conserved across nematode species, it does not necessarily follow that they control sex determination in a particular species, since several genes in the *Caenorhabditis* sex determination pathway are pleiotropic and have functions outside sex determination. Future functional studies will be required to determine whether conserved genes indeed regulate sex determination in species distant from *Caenorhabditis* and whether other genes have been recruited to the sex determination pathway in these species. Together, these findings suggest that the mechanisms and genes controlling nematode sex determination may be more varied than previously appreciated.

## Supplementary Information

Supplementary Dataset 1.

Supplementary Dataset 2.

Supplementary Dataset 3.

Supplementary files.

## Data Availability

Raw RNA-Seq reads for free-living adult males are available in the NCBI SRA database under BioProject ID: PRJNA689252.
