# Supplementary material for: Transcriptional profiles in Strongyloides stercoralis males reveal deviations from the Caenorhabditis sex determination model
Source: Sci Rep. 2021 Apr 15;11:8254. doi: 10.1038/s41598-021-87478-3 (PMC8050236; doi:10.1038/s41598-021-87478-3)
Supplement: Supplementary file 4 — Supplementary files. [file 41598_2021_87478_MOESM4_ESM.pdf]

SUPPLEMENTARY TABLES AND FIGURES

Transcriptional profiles in *Strongyloides stercoralis* males reveal deviations from the *Caenorhabditis* sex determination model

Damia Gonzalez Akimori[1], Emily J. Dalessandro[1], Thomas J. Nolan[2], Christopher R. Stieha[1], James B. Lok[2], and Jonathan D.C. Stoltzfus[1*]

[1]Department of Biology, Millersville University of Pennsylvania, Millersville, Pennsylvania 17551, USA.
[2]Department of Pathobiology, School of Veterinary Medicine, University of Pennsylvania, Philadelphia, Pennsylvania 19104, USA.
*Corresponding author. Email: Jonathan.Stoltzfus@millersville.edu

Supplemental Table S1. Male-specific predicted single-pass transmembrane proteins clustered on scaffold 5.

Gene Name	Predicted number of amino acids	Predicted signal peptide likelihood	Predicted transmembrane domain
SSTP_0000016300	72	0.0011	20-42 aa
SSTP_0000016400	67	0.0003	23-45 aa
SSTP_0000016500	161	0.0005	18-40 aa
SSTP_0000016600	161	0.0007	18-40 aa
SSTP_0000016650*	60	0.0062	10-32 aa
SSTP_0000016700	110	0.0014	10-32 aa
SSTP_0000016800	70	0.0016	15-37 aa
SSTP_0000016900	150	0.0004	12-34 aa
SSTP_0000016950*	57	0.0002	10-32 aa
SSTP_0000017010**	178	0.0026	---
SSTP_0000017020*	59	0.0005	18-40 aa
SSTP_0000017030**	148	0.0178	18-40 aa

*Previously unannotated; **Previously SSTP_0000017000 (annotation included two genes).

Supplemental Table S2. Male-specific predicted single-pass transmembrane proteins clustered on scaffold 9.

Gene Name	Predicted number of amino acids	Predicted signal peptide likelihood	Predicted transmembrane domain
SSTP_0001266600	126 aa	0.7582	102-124 aa
SSTP_0001267100	100 aa	0.8637	75-97 aa
SSTP_0001267200	122 aa	0.8293	70-91 aa
SSTP_0001267300	162 aa	0.9944	--
SSTP_0001267400	190 aa	0.9612	95-117 aa
SSTP_0001267500*	193 aa	0.9964	172-191 aa
SSTP_0001267600	180 aa	0.0022	114-136 aa
SSTP_0001267700	105 aa	0.5341	69-91 aa
SSTP_0001267800	209 aa	0.9972	188-207 aa
SSTP_0001267900	189 aa	0.9966	168-187 aa
SSTP_0001268000	219 aa	0.0041	125-147 aa
SSTP_0001268100	182 aa	0.9876	158-180 aa
SSTP_0001268210**	190 aa	0.9956	--
SSTP_0001268220**	165 aa	0.9964	--
SSTP_0001268300 **(pseudo/truncated gene)**	n/a	n/a	n/a

*5’ end was corrected; **Previously SSTP_0001268200 (annotation included two genes).

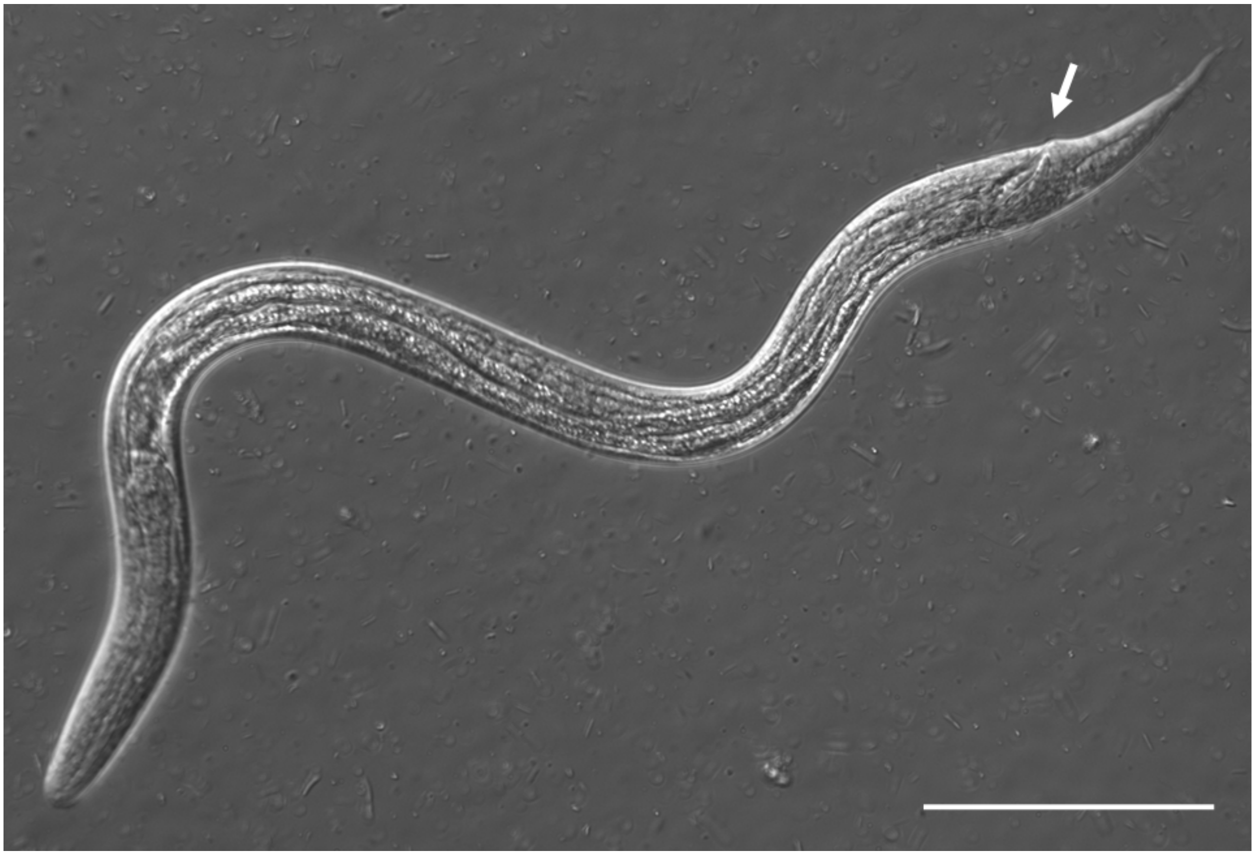

Supplemental Figure S1. Precociously developing rhabditiform *S. stercoralis* male from a permissive host undergoing autoinfection.

Rhabditiform late L4/adult male recovered from the large intestine of an experimentally infected Mongolian gerbil (*Meriones unguiculatus*) treated with corticosteroids and undergoing autoinfection. Arrow denotes the pair of copulatory spicules, which are only present in males. Scale bar is 100 μm.

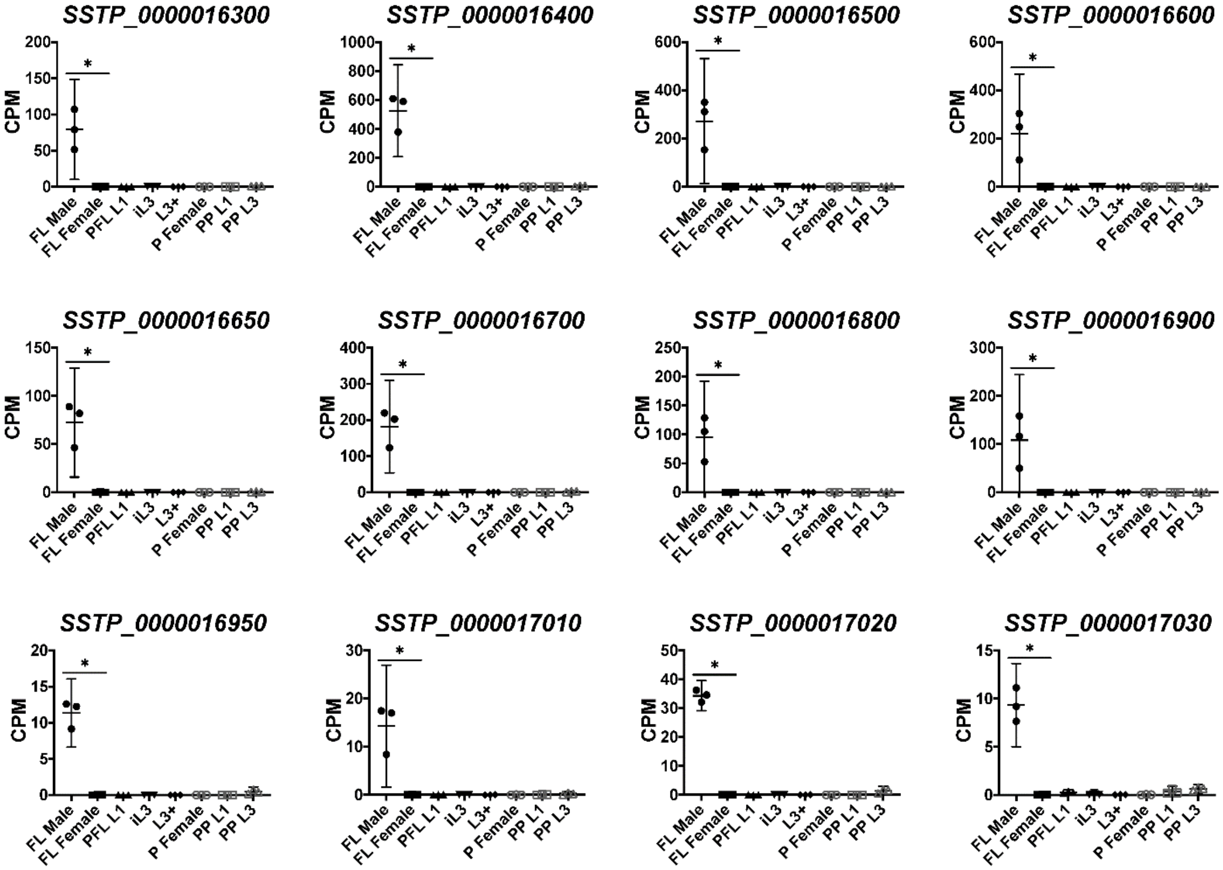

Supplemental Figure S2. Transcripts originating from a cluster of genes on scaffold 5 are up-regulated in free-living adult males.

Transcript abundances of 12 genes clustered on *S. stercoralis* scaffold 5 are represented using TMM-normalized counts per million (CPM) for the following developmental stages: free-living adult males (FL Male), free-living gravid adult females (FL Female), post-free-living first-stage larvae (PFL L1), developmentally arrested infectious third-stage larvae (iL3), L3 activated inside a permissive host (L3+), parasitic gravid adult females (P Female), heterogonically-developing post-parasitic L1 (PP L1), and heterogonically-developing post-parasitic L3 enriched for females (PP L3). Graphs were constructed using GraphPad Prism v.9.0.0; bars indicate means (horizontal) and 95% confidence intervals (vertical) for each of the three biological replicates represented as individual data points. Asterisks (*) indicate a significant (fold change >2.0; FDR <0.05) difference between FL Male and FL Female.

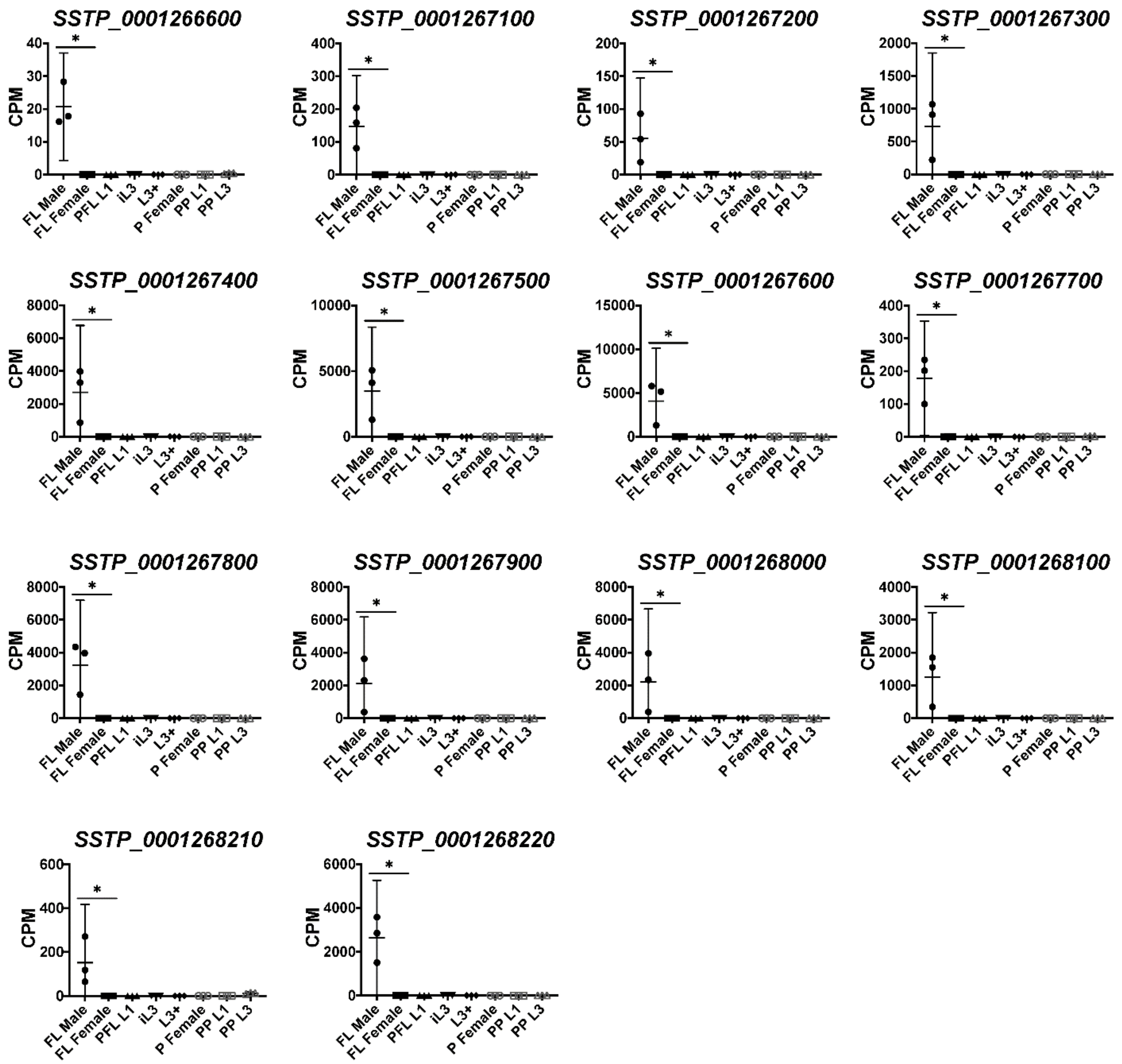

Supplemental Figure S3. Transcripts originating from a cluster of genes on scaffold 9 are up-regulated in free-living adult males.

Transcript abundances of 14 genes clustered on *S. stercoralis* scaffold 9 are represented using TMM-normalized counts per million (CPM) for the following developmental stages: free-living adult males (FL Male), free-living gravid adult females (FL Female), post-free-living first-stage larvae (PFL L1), developmentally arrested infectious third-stage larvae (iL3), L3 activated inside a permissive host (L3+), parasitic gravid adult females (P Female), heterogonically-developing post-parasitic L1 (PP L1), and heterogonically-developing post-parasitic L3 enriched for females (PP L3). Graphs were constructed using GraphPad Prism v.9.0.0; bars indicate means (horizontal) and 95% confidence intervals (vertical) for each of the three biological replicates represented as individual data points. Asterisks (*) indicate a significant (fold change >2.0; FDR <0.05) difference between FL Male and FL Female.

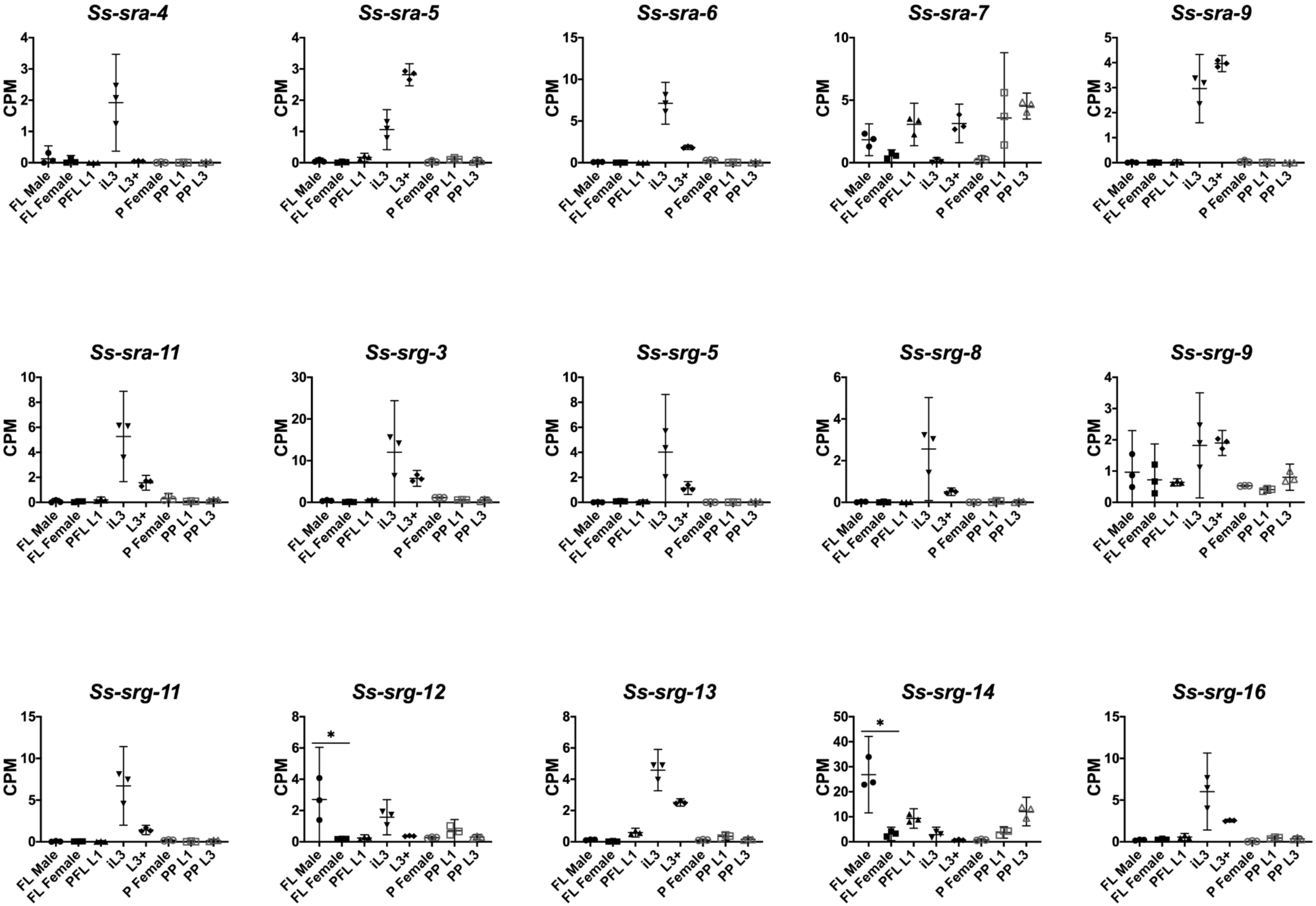

Ss-sra-4
Ss-sra-5
Ss-sra-6
Ss-sra-7
Ss-sra-9
Ss-sra-11
Ss-srg-3
Ss-srg-5
Ss-srg-8
Ss-srg-9
Ss-srg-11
Ss-srg-12
Ss-srg-13
Ss-srg-14
Ss-srg-16
CPM
FL Male
FL Female
PFL L1
iL3
L3+
P Female
PP L1
PP L3

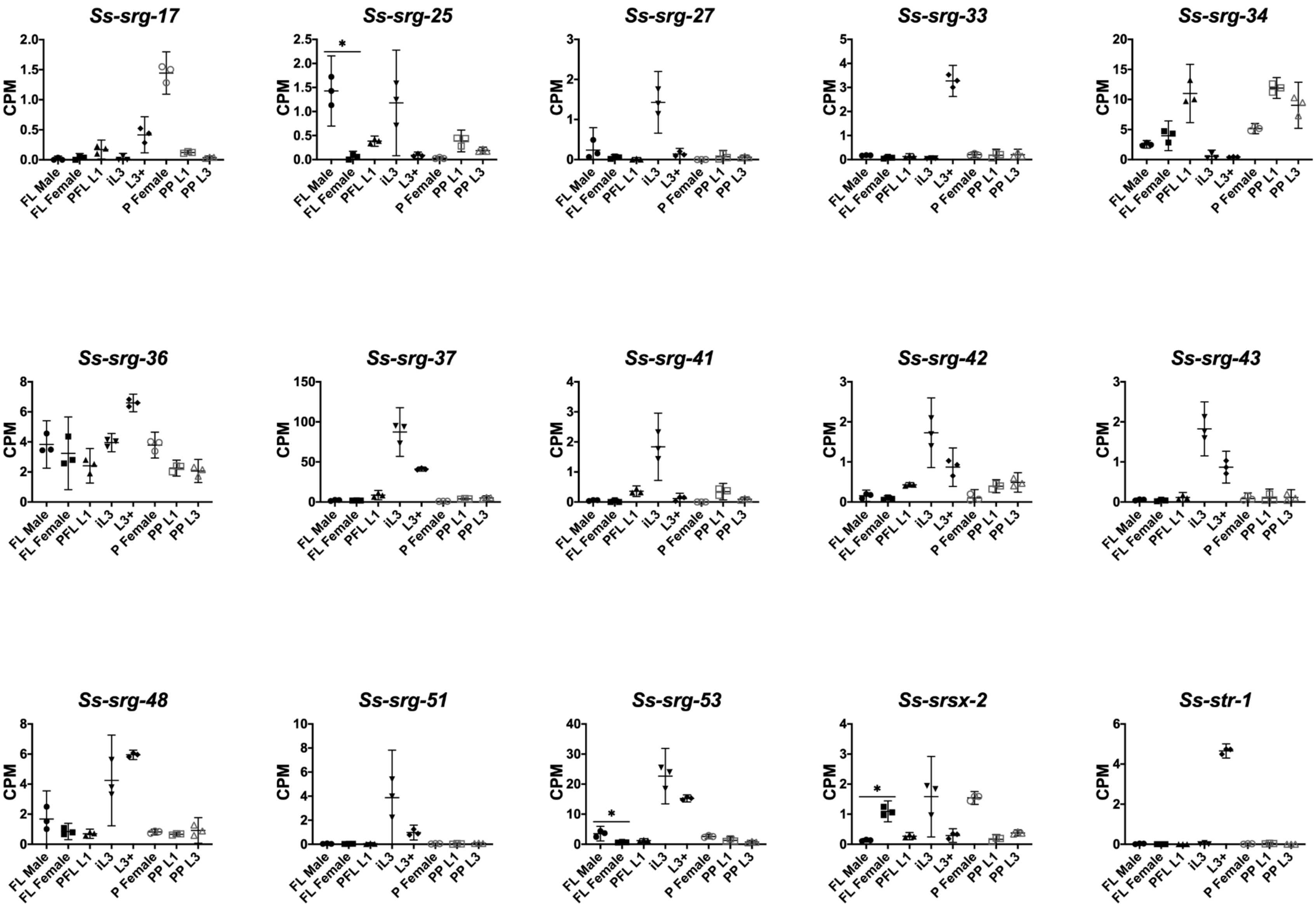
Ss-srg-17
Ss-srg-25
Ss-srg-27
Ss-srg-33
Ss-srg-34
Ss-srg-36
Ss-srg-37
Ss-srg-41
Ss-srg-42
Ss-srg-43
Ss-srg-48
Ss-srg-51
Ss-srg-53
Ss-srsx-2
Ss-str-1
CPM
*
FL Male
FL Female
PFL L1
iL3
L3+
P Female
PP L1
PP L3

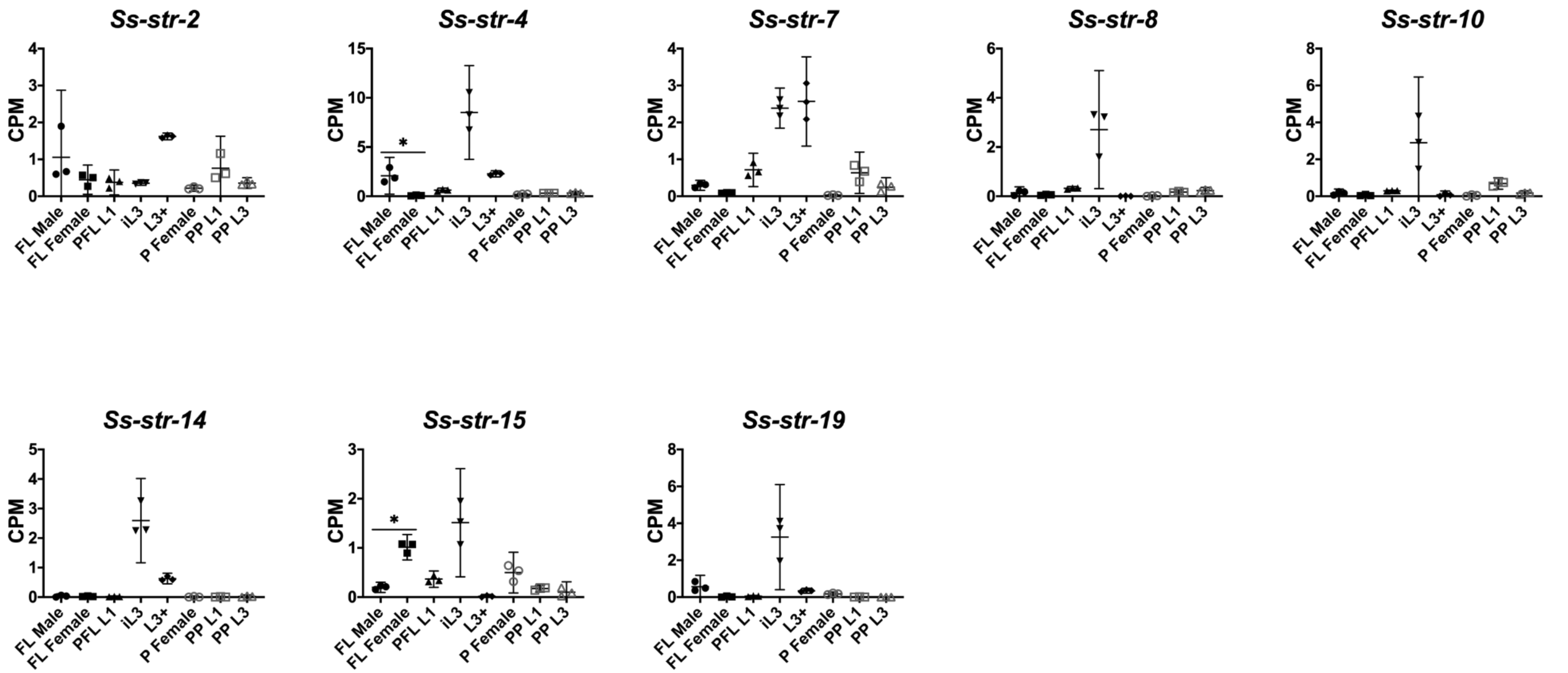

Supplemental Figure S4. Transcripts encoding putative G protein-coupled receptors are differentially expressed during *S. stercoralis* development.

Transcript abundances of 38 genes encoding putative G protein-coupled receptors are represented using TMM-normalized counts per million (CPM) for the following developmental stages: free-living adult males (FL Male), free-living gravid adult females (FL Female), post-free-living first-stage larvae (PFL L1), developmentally arrested infectious third-stage larvae (iL3), L3 activated inside a permissive host (L3+), parasitic gravid adult females (P Female), heterogonically-developing post-parasitic L1 (PP L1), and heterogonically-developing post-parasitic L3 enriched for females (PP L3). Graphs were constructed using GraphPad Prism v.9.0.0; bars indicate means (horizontal) and 95% confidence intervals (vertical) for each of the three biological replicates represented as individual data points. Asterisks (*) indicate a significant (fold change >2.0; FDR <0.05) difference between FL Male and FL Female.

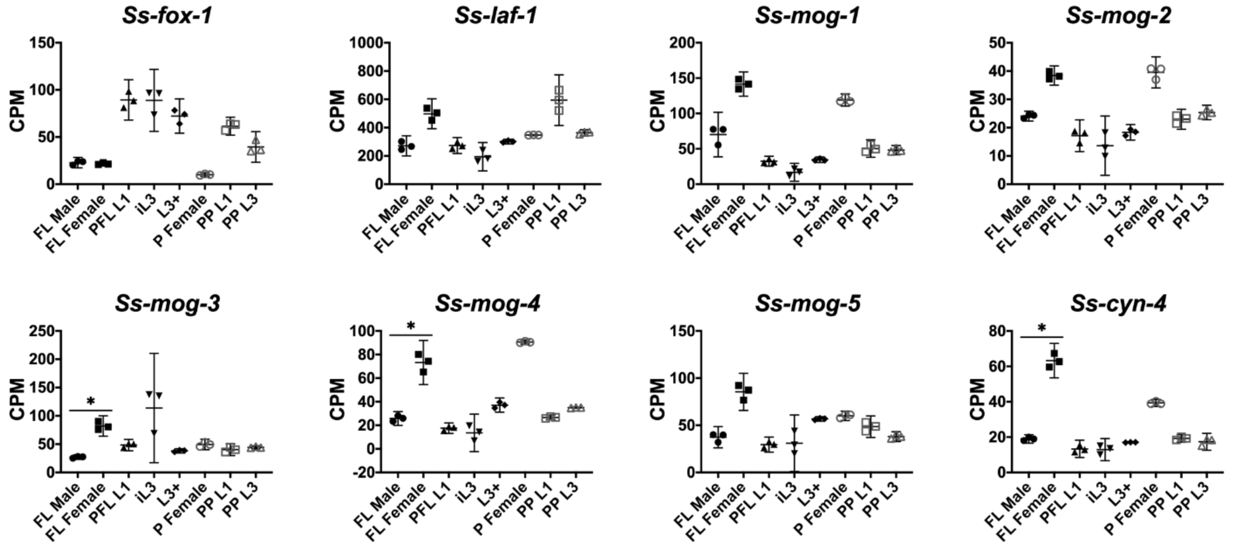

Supplemental Figure S5. Transcript abundance of *S. stercoralis* homologs of genes involved in *Caenorhabditis* sex determination.

We identified *S. stercoralis* homologs of *fox-1* (predicted to encode an RNA-binding protein), *laf-1* (predicted to encode a DEAD box helicase), *mog-1* (predicted to encode a DEAD box helicase), *mog-2* (predicted to encode the small nuclear ribonucleoprotein polypeptide A'), *mog-3* (predicted to encode the spliceosome-associated protein CWC25), *mog-4* (predicted to encode a DEAD box helicase), *mog-5* (predicted to encode a DEAD box helicase), and *cyn-4* (predicted to encode a ubiquitin ligase; formerly *mog-6*) by reciprocal BLAST searches. Transcript abundances of *S. stercoralis* homologs are represented using TMM-normalized counts per million (CPM) for the following developmental stages: free-living adult males (FL Male), free-living gravid adult females (FL Female), post-free-living first-stage larvae (PFL L1), developmentally arrested infectious third-stage larvae (iL3), L3 activated inside a permissive host (L3+), parasitic gravid adult females (P Female), heterogonically-developing post-parasitic L1 (PP L1), and heterogonically-developing post-parasitic L3 enriched for females (PP L3). Graphs were constructed using GraphPad Prism v.9.0.0; bars indicate means (horizontal) and 95% confidence intervals (vertical) for each of the three biological replicates represented as individual data points. Asterisks (*) indicate a significant (fold change >2.0; FDR <0.05) difference between FL Male and FL Female.

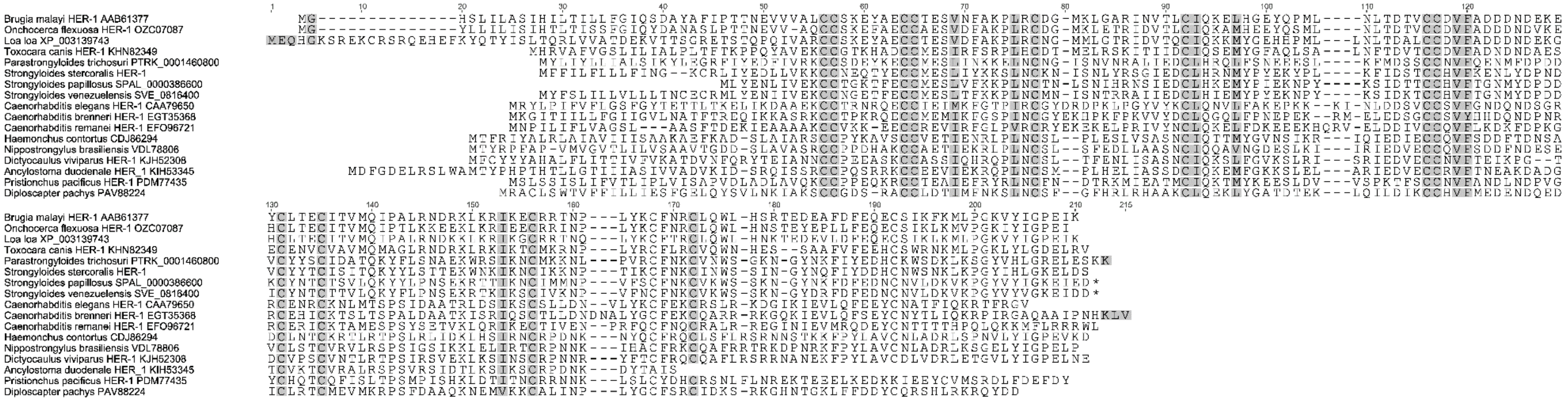

Supplemental Figure S6. Protein alignment of nematode HER-1 homologs.

We identified homologs of *C. elegans* HER-1 by reciprocal BLAST searches in clade III, IV, and V nematodes. We aligned predicted polypeptides using CLUSTAL and highlighted conserved residues in Geneious v.11.1.5. Accession numbers and gene names are listed after the species names.

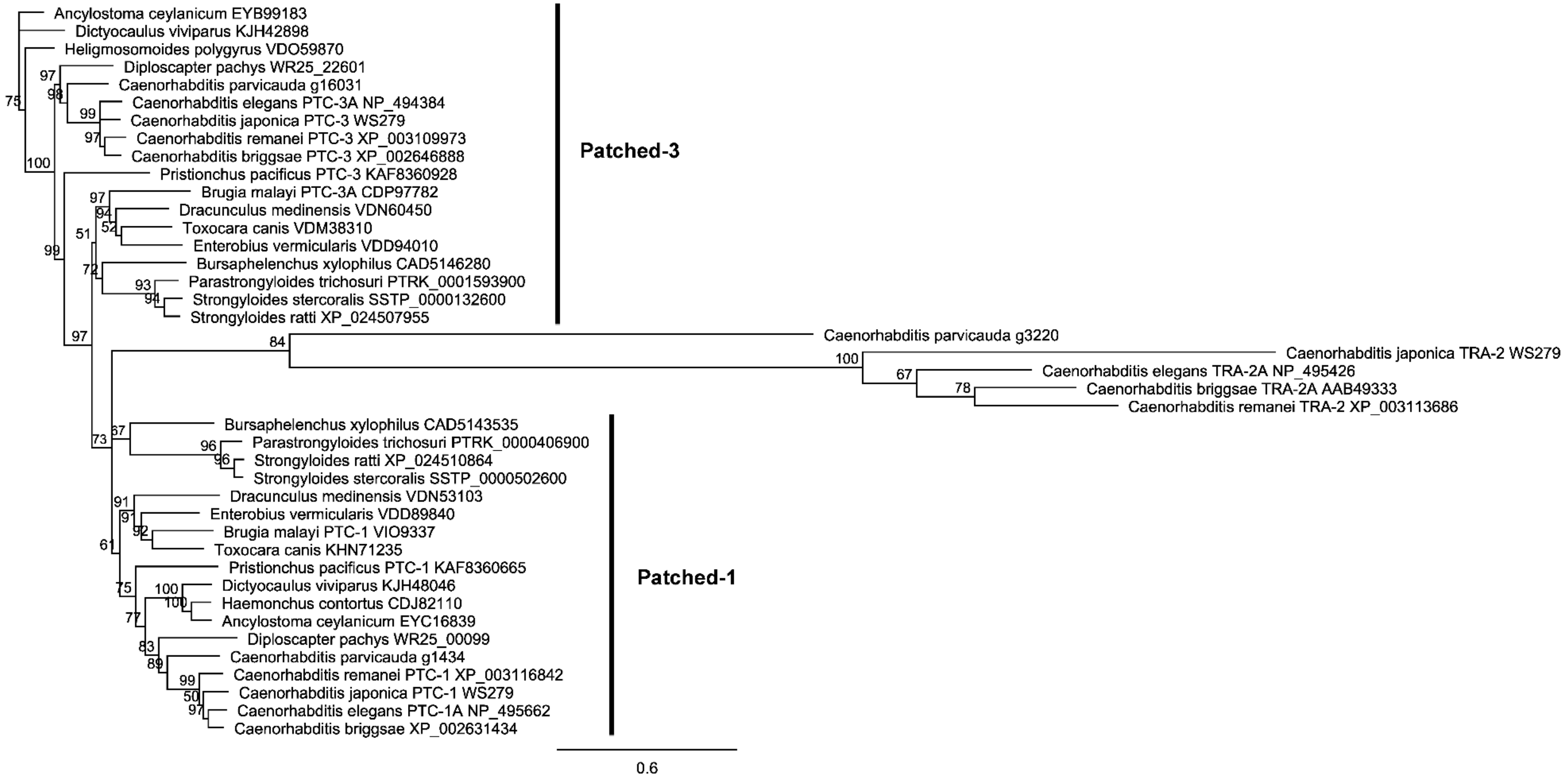

Supplemental Figure S7. TRA-2 is a highly divergent homolog of Patched-1 and Patched-3.
We identified homologs of Patched-1 and Patched-3 in clade III, IV, and V nematodes using reciprocal BLAST searches. We were only able to identify TRA-2 homologs in *Caenorhabditis* species. We aligned the predicted polypeptide sequences using CLUSTAL and created a neighbor-joining phylogenetic tree with 100 iterations of bootstrapping using Geneious v.11.1.5. Accession numbers and gene names are listed after the species names.
